# Confronting the anxiety of Generation Z: electroacupuncture therapy regulates oxidative stress and microglia activity in amygdala-basolateral of socially isolated mice

**DOI:** 10.3389/fpsyt.2024.1496201

**Published:** 2025-02-06

**Authors:** Tong Yin, Junyun Yuan, Lu Liu, Yinxin Wang, Yuanfang Lin, Kangwen Ming, Hang Lv

**Affiliations:** ^1^ Guangzhou University of Chinese Medicine, Guangzhou, China; ^2^ Department of Tuina, The Fourth Clinical College of Guangzhou University of Chinese Medicine, Shenzhen, China; ^3^ Department of Acupuncture and Rehabilitation, The Affiliated Traditional Chinese Medicine Hospital of Guangzhou Medical University, Guangzhou, China

**Keywords:** Generation Z, social isolation, anxiety disorder, electroacupuncture, microglia, basolateral amygdala, oxidative stress

## Abstract

**Introduction:**

Anxiety disorders are prevalent mental health conditions characterized by significant impairments in daily functioning and social interactions. Despite the effectiveness of pharmacological treatments, challenges such as medication resistance, adverse side effects, and the high rate of relapse necessitate the exploration of alternative therapies. Recently, electroacupuncture (EA) has garnered attention as a promising non-pharmacological intervention for anxiety disorders; however, the mechanisms by which EA exerts its anxiolytic effects remain poorly understood. This study aims to elucidate the role of microglial cells in anxiety, specifically examining how EA modulates microglial morphology and function within the basolateral amygdala (BLA) in the context of anxiety induced by social isolation.

**Methods:**

Utilizing a mouse model of social isolation-induced anxiety, we evaluated anxiety-like behaviors through the Elevated Plus Maze (EPM) and Open Field Test (OFT). Additionally, biochemical analyses and immunofluorescence imaging were performed to assess the expression of NADPH oxidase 2 (NOX2), microglial activation markers, and levels of oxidative stress.

**Results:**

Our findings reveal that EA treatment significantly mitigates anxiety-like behaviors in mice, correlating with a reduction in NOX2 expression within BLA microglia and decreased levels of reactive oxygen species (ROS). Furthermore, EA was observed to restore normal microglial morphology, indicating its potential role in modulating microglial activity.

**Discussion:**

The results of this study suggest that EA exerts its anxiolytic effects through the modulation of oxidative stress and the activity of microglia in the BLA. These findings provide new insights into the cellular mechanisms underlying the therapeutic effects of EA, highlighting the potential for non-pharmacological strategies in the management of anxiety disorders and paving the way for future research aimed at improving clinical outcomes for individuals suffering from anxiety.

## 1 Introduction

Anxiety is a psychological state that can cause excessive fear and avoidance behaviors due to anticipation of threats, affecting brain structure and function (1). Multiple anxiety disorders, including social anxiety disorder, are common and disabling, significantly impacting quality of life and social functioning. Social anxiety disorder affects an estimated 13% of individuals and 4.05% of the global population, posing a threat to well-being. Treatment for anxiety disorders, which accounts for over 50% of global mental health expenditure, relies heavily on medication and adds to social and economic burdens (2, 3). SSRIs and SNRIs are effective initial treatments for the disorder, but may encounter challenges like resistance, compliance, and social/economic/cultural factors (3). Drug therapy has limitations like low effectiveness, side effects, slow results, and high chances of recurrence. Cognitive Behavioral Therapy (CBT) is a non-pharmacological option that targets the underlying causes of anxiety, but needs ongoing maintenance (3, 4). More patients are opting for complementary and alternative therapies, such as relaxation, nutritional supplements, massage, and acupuncture, to avoid medication side effects (5). Research has found that acupuncture can be effective in treating chronic anxiety disorders, especially when traditional treatments fail (6). Reports suggest that acupuncture, alone or in combination with medication, can help treat anxiety disorders; however, the exact mechanisms are not fully understood (7, 8).

In recent years, numerous randomized controlled trials (RCTs) and systematic reviews have demonstrated the efficacy of electroacupuncture in treating anxiety disorders. For instance, a double-blind RCT conducted by Amorim et al. found that electroacupuncture significantly reduced anxiety symptoms, with the treatment group showing a marked decrease in anxiety scores compared to the control group (9). Additionally, a systematic review by Li et al. compiled clinical studies on electroacupuncture for anxiety, confirming its effectiveness not only in alleviating anxiety but also in enhancing patients’ quality of life (7). Research on acupoints such as GV20 (Baihui) and GV29 (Yintang) has shown that stimulation at these sites can modulate brain regions involved in emotion and stress response, including the amygdala, prefrontal cortex, and hypothalamus, thereby reducing oxidative stress and regulating neural function (10, 11). These studies collectively support the clinical application of electroacupuncture, particularly at GV20 and GV29, and highlight its potential therapeutic benefits for anxiety management.

Social isolation, limited social connections, and loneliness are closely linked to social anxiety disorders, impacting emotional regulation, physical health, and overall well-being (12). Social isolation used to mainly impact elderly people, children left behind disabled individuals, low-income earners, and immigrants due to factors like limited resources, movement restrictions, language barriers, and loss of peers (13). However, Generation Z (Gen Z), who born between 1997 and 2012, grew up heavily influenced by social media compared to other generations (14). While social media helps Gen Z communicate, studies have shown that it can harm mental health (15, 16). Research has shown that social media can cause feelings of loneliness and social exclusion, leading to social anxiety and isolation in Gen Z (17, 18). Therefore, we used a social isolation mouse anxiety model to study the impact of Gen Z social isolation on social anxiety disorder (19). This model improves our knowledge of how social isolation leads to social anxiety symptoms and provides direction for future research and treatment.

The basolateral amygdala (BLA) neurons play a crucial role in emotional regulation, being highly responsive to external stimuli and receiving input from different brain regions (20). The BLA plays a crucial role in emotional reactions and stress regulation by integrating sensory inputs from various brain regions and modulating anxiety behaviors and physiological responses (21). The amygdala and hippocampus work together to regulate emotional responses, with the hippocampus aiding the amygdala in recognizing and responding to stress (22). Studies on animal models showed that activating the neural pathway between the BLA and vCA1 in mice can decrease anxiety levels during certain tasks (23). The mPFC plays a key role in controlling AMY activity to prevent overreacting emotionally. If this process is disrupted, it can lead to increased connectivity between the mPFC and AMY, potentially causing maladaptive responses and anxiety symptoms under prolonged stress (24). Scholarly studies have demonstrated that the BLA can detect threat signals from the environment and control the body’s natural cannabinoid signaling, particularly 2-AG, which is involved in managing stress and adapting to it (25). Chronic stressors like social isolation activate the BLA, which in turn activates the HPA axis, causing stress responses like inflammation and metabolic issues that can contribute to anxiety and psychiatric disorders (26, 27). Studying the BLA with optogenetic and chemogenetic methods has shown that changing its activity can cause anxiety-related behaviors (28, 29). Electrophysiological recordings show increased BLA neuronal activity in response to anxiety-provoking stimuli, correlating with observed anxiety behaviors (30). fMRI studies show higher BLA activity in people with anxiety disorders compared to healthy individuals, and this activity is positively linked to the severity of anxiety symptoms (31). Studies on lesions in the BLA have shown that damage to this region results in decreased anxiety-related behaviors (32). These results highlight the role of BLA in anxiety regulation and its interactions with other brain regions. Further research on the BLA is crucial for understanding anti-anxiety treatments.

The brain’s high oxygen consumption makes it vulnerable to damage from oxidative stress (33). Oxidative stress can disrupt cell membranes, proteins, and DNA, leading to neuronal dysfunction and cell death (34). Oxidative stress can damage regions important for emotions and memory, like the BLA, by activating microglia and releasing pro-inflammatory cytokines. This affects neuronal function, synaptic plasticity, and signal transmission (35), potentially triggering anxiety symptoms. Furthermore, research indicated that oxidative stress in the AMY/BLA region can trigger anxiety symptoms, emphasizing its significance in anxiety mechanisms (36).

NOX2, an enzyme in the nervous system, is a primary source of oxidative stress, producing superoxide anions and hydrogen peroxide that are pivotal in cellular signaling and immune response (37). Chronic stress, like social isolation, boosts NOX2 levels in microglia, causing the release of ROS and potentially leading to neurodegeneration and oxidative stress (35). Studies have demonstrated that the overactivity of NOX2 results in excessive production of ROS, causing oxidative damage within the nervous system. This damage not only induces pathological processes such as neuronal dysfunction, synaptic toxicity, and cell death, but also disrupts the balance between excitatory and inhibitory signals within neurons (34, 36). Moreover, the upregulation of NOX2 under stress conditions is closely associated with changes in anxiety behaviors (38). Therefore, NOX2 is a pivotal factor in oxidative stress within the nervous system, and its activity leads to oxidative damage and neurostressor responses, thereby affecting neuronal function and anxiety behaviors.

Microglia, as critical immune cells of the central nervous system, remain highly active in a resting state, monitoring potential risks within the brain and responding sensitively to ROS signals, thus participating in neuroinflammatory processes (37). Microglia quickly release pro-inflammatory cytokines and oxidants like hydrogen peroxide to protect neurons when they detect pathogens or cellular damage. NOX2 expression in microglia makes them important regulators of oxidative stress in the brain (35). Under chronic stress conditions, the upregulation of NOX2 further activates microglia, enhancing ROS production and release, leading to exacerbated neuroinflammation and oxidative stress. Additionally, the persistent presence of oxidative stress and microglial activation results in a cycle of neuronal damage and inflammatory responses, thereby promoting anxiety and other psychiatric disorders under chronic pathological states (39). Thus, the upregulation of NOX2 and oxidative stress may be closely related to microglial activation.

This study aims to bridge the current knowledge gap by examining the effects of EA on microglial morphology and function in the BLA, a critical brain region for anxiety regulation. We propose a mechanism by which electroacupuncture may modulate oxidative stress and microglial activity induced by social isolation. Employing a mouse model of anxiety induced by social isolation, we evaluated the expression of NOX2, microglial activation, and oxidative stress markers within the BLA. This was done to clarify the involvement of microglia in the anxiolytic effects of EA, to provide novel insights into the cellular mechanisms of EA, and to emphasize its potential for modulating neuroinflammation and oxidative stress in the context of anxiety disorders.

## 2 Method

### 2.1 Animals and materials

Although studies suggest that rats might be more sensitive to social isolation (40), research also indicates that social isolation can induce neuroinflammation and anxiety-like behavioral changes in mice (41). Therefore, we involved Male C57BL/6 mice (8-10 weeks, 20–25 g) obtained from Guangzhou ZhiYuan Biopharmaceutical Technology Co., Ltd in this study. They were randomly assigned to groups and kept in a standard laboratory environment with controlled temperature, humidity, bedding material, food, water, and light/dark cycle. The animal experimental procedures adhered to the approved protocol of the Animal Care and Use Committee of Guangzhou University of Traditional Chinese Medicine and were in accordance with the ARRIVE guidelines and the Guide for the Care and Use of Laboratory Animals, as recommended by the National Institutes of Health. A seven-day acclimation period to the housing environment was provided for the animals prior to the commencement of the experiment.

### 2.2 Antibodies

The Western Blot antibodies used in this study included Mouse anti-gp91 antibody (BDB611414) from BDbioscien (USA) and Mouse anti-β-ACTIN antibody (AF5003) and HRP-conjugated goat anti-mouse secondary antibody (AF0208) from Beyotime Biotech (China). The Immunofluorescence antibodies used were mouse anti-gp91 antibody (BDB611414) from BDbioscien (USA), rabbit anti-IBA1 antibody (019-19741) from Wako (Japan), CoraLite488-labeled goat anti-mouse IgG(H+L) (SA00013-1), and CoraLite594-labeled goat anti-rabbit IgG(H+L) (SA00013-4) from Proteintech (China).

### 2.3 Social isolation

Multiple studies have reported that social isolation can lead to the development of anxiety- and depression-like behaviors in rodents (19, 42).

In this study, a total of 48 male C57BL/6 mice aged 8-10 weeks and weighing 20-25 g were utilized, with 12 mice allocated to each group. Following a 7-day acclimatization period to the new environment, mice in the social isolation group were individually housed for 4 weeks, while those in the control group were housed in groups of 4-5 mice (43, 44). The experimental procedures involved no human intervention, except for weekly cage changes. The groups were delineated as follows:

1. The control group consisted of mice housed in groups of four per cage for a duration of four weeks, with ad libitum access to water and food, followed by anesthesia administered after four weeks.
2. The control group with EA treatment consisted of mice housed in groups of four per cage for a duration of four weeks, with ad libitum access to water and food. Anesthesia was administered after four weeks, followed by EA treatment.
3. The social isolation group consisted of mice housed individually for a duration of four weeks, with ad libitum access to water and food, followed by anesthesia administered after four weeks.
4. The social isolation group with EA treatment consisted of mice housed individually for a duration of four weeks, with ad libitum access to water and food. Anesthesia was administered after four weeks, followed by EA.

### 2.4 Electroacupuncture

EA treatment began 2 days after the end of the social isolation period and lasted for 4 weeks. The intervention treatment was consistently administered at a set time each day, usually within the standard nighttime activity period (8 pm-10 pm). The EA treatment room was arranged 30 min before treatment initiation to allow the mice to acclimate to their surroundings. The EA interventions were conducted by experienced animal care personnel utilizing isoflurane anesthesia administered through a gas anesthesia machine (3% isoflurane in RWD Medical Co., Ltd., Shenzhen, China) at a flow rate of 1.0-1.5 L/min for induction of anesthesia, followed by adjustment to a concentration of 1.5% isoflurane at a flow rate of 0.4-0.8/min for maintenance of anesthesia during the entirety of the procedure. The level of anesthesia was assessed through monitoring hind limb muscle tone and body twitching, with modifications made to maintain appropriate anesthesia levels and reduce discomfort. Following stabilization of the anesthesia, sterile needles measuring 0.25 mm in diameter and 13 mm in length were inserted horizontally into the GV20 and GV29 acupoints on the mouse’s head, which correspond to anatomical sites traditionally utilized for treating emotional disorders in humans (43, 44). The EA current was set at 1 mA and applied at a frequency of 2 Hz using a HANS stimulator (HANS-200A/100 B, Beijing, China) for 30 minutes, with treatments administered on alternate days. Mice in the control group were anesthetized with isoflurane.

### 2.5 Anxiety behavior test

The elevated plus maze (EPM) and open field test (OFT) are commonly used to assess anxiety in rodents. Anxiety testing occurred 24h after treatment. Because mice are more active at night, testing took place between 8 pm and midnight to ensure accurate results and minimize circadian effects.

Open Field Test (OFT)Prior to the experiment, mice were acclimatized in the testing room for 1 h every 3 days. During the experiment, the mice were placed in a 45 cm × 45 cm × 45 cm cubic testing box with intense light on a 25 cm × 25 cm central area. After 10 min of free exploration, the mice were placed in the next group. Droppings were cleared, and 75% ethanol was used to sanitize and eliminate residual odors after each trial. LabState software was used to analyze the time and distance covered by the animals in the central area during testing.

### 2.6 Elevated plus maze

The elevated plus maze test was conducted according to a standard protocol in a dedicated acoustically isolated room with consistent lighting. The maze had a “+” configuration with two open arms and two closed arms forming an enclosed square. It was located 50 cm above the floor. If a mouse fell, the trial was restarted after the previous round. Each animal was placed in a 5X5cm square facing the open arm, and their 5-minute exploration was recorded. After each trial, the excrement was removed, and olfactory cues were neutralized using 75% ethanol. The Any-maze software (Shanghai Xiansoft Information Technology Co., Ltd.) was used to analyze the time spent on the open arms.

### 2.7 DHE experiment

Oxidative stress was evaluated using two approaches: (1) reactive oxygen species (ROS) levels were quantified using dihydroethidium (DHE) staining, a fluorescent probe that detects superoxide anions and is widely used to assess oxidative stress (45)^;^ (2) lipid peroxidation was measured by malondialdehyde (MDA) detection, and antioxidant capacity was determined through superoxide dismutase (SOD) activity assessment (43). To avoid any interference with the expression levels of DHE, the mice were placed in a quiet environment without any disturbance before intraperitoneal injection. DHE was dissolved in DMSO and diluted to 0.1 mg/ml with physiological saline. A dose of 2 mg/kg was administered intraperitoneally. After an hour, the mice were anesthetized, perfused with PBS, and their brains were extracted. Brain tissue was embedded in OCT, sectioned into 10μm slices, and mounted on glass slides. After drying, the slices were washed with PBS for 15 min and immersed in a glycerol:PBS solution for coverslip application. The specimens were then sealed and subjected to fluorescence imaging.

### 2.8 Fluorescence detection

Laser scanning confocal microscopy was used to detect fluorescence at 594 nm on brain slides. Fluorescence images were then acquired. Three to five brain slices per region of interest were chosen. The mean fluorescence intensity was calculated from three to four slices of three to five mice using the ImageJ software. Subsequently, data standardization and processing were conducted.

### 2.9 MDA/SOD detection

The MDA/SOD content in the mouse amygdala tissue was measured using an MDA detection kit (A003-1, Nanjing Jiancheng) according to the manufacturer’s instructions. Amygdala tissue was extracted and homogenized in lysis buffer after anesthesia and collection. The samples were incubated with the reagents at 95°C for 30 min. After centrifugation, the absorbance was measured at 532 nm to calculate the MDA content using a UV spectrophotometer.

### 2.10 Western blot analysis

Before performing the western blot experiment, sterilized Eppendorf tubes were partitioned and weighed for tissue weight calculations. Tissue samples were collected and separated in Eppendorf tubes under refrigeration. RIPA lysis buffer and a mixture of protease and phosphatase inhibitors were added to the tubes. The tissue was lysed for 30 min, homogenized with an ultrasonic processor, and centrifuged at 4°C for 15 min. Protein concentration was measured using a BCA protein assay kit. SDS-PAGE protein loading buffer was added and heated. Proteins were separated on an 8% Gly SDS-PAGE gel. The proteins were then transferred to PVDF membranes. Membranes were washed with TBST solution and blocked with a quick blocking buffer. Primary antibodies against gp91 and ACTIN were used for immunostaining. The membrane was washed and incubated with the secondary antibody. After rinsing, the membrane was treated with ECL solution and protein blotting was performed using a chemiluminescence system.

### 2.11 Enzyme linked immunosorbent assay

An ELISA was used to measure NOX levels in the tissues using a detection kit. The tissue was mixed with reagents according to the manufacturer’s instructions and disrupted using ultrasound. After disruption, the tissue was centrifuged at 4°C and 600 × g for 5 min to extract the supernatant, which was then centrifuged at 4°C and 11000 × g for 10 min to obtain the cytosolic extract. The precipitate was mixed with reagents 2 and 3 from the kit, shaken repeatedly, and added to the measurement and control tubes in a 96-well plate according to the manufacturer’s instructions (Solarbio, China). The initial absorbance was recorded at 600 nm for 20 s using a microplate spectrophotometer (BioTek, USA).

### 2.12 Immunofluorescence chemical analysis

Brain tissue was embedded in Tissue-Tek OCT and sliced using a freezing microtome. The slices were stored in a freezing solution at -20°C. After staining, the slices were washed with PBS and blocked with a solution containing BSA and Triton X-100. The primary antibodies, anti-Nox2 and anti-Iba1, were diluted in blocking solution and incubated with the slices overnight. The following day, the slices were washed with PBS containing Tween 20 and incubated with secondary antibodies. After incubation, the slices were washed with PBS-T and mounted onto microscope slides with DAPI. The slices were then washed with PBS-T, air-dried, sealed with glycerol and PBS solution, and stored at -20°C. Fluorescence imaging was performed using a laser confocal microscope.

### 2.13 Confocal microscope fluorescent imaging

When inflammation, injury, or other conditions occur, there may be changes in the number of microglia. Quantitative techniques for assessing microglial morphology provide precise depictions (46, 47). This study used laser-scanning confocal microscopy to capture Z-stack images at 20x magnification with consistent settings (40µm z stack, interval 2µm, Nikon A1). The experimental design included four groups with three animals each. Brain slices were obtained from specific BLA regions, and four slices were randomly selected for imaging. Any slices containing impurities were replaced. This resulted in 48 images showing the colocalization of gp91 and IBA1. Fiji (ImageJ) and plugins were used for analysis. The study focused on microglial activation and further investigated the relationship with EA intervention using 40x imaging in AMY-BLA with consistent parameters. A total of 36 images were captured and analyzed using Fiji software with suitable plugins.

### 2.14 Skeletal analysis of the microglia cells

Appropriate parameters were set in the filter menu. The despeckle process removed the salt-and-pepper noise caused by the blurred mask. The image was converted to a binary format by adjusting the threshold settings. Before the application, a threshold value was chosen. Despeckle, close, and outlier functions were used to reduce background noise. Despeckle accessed via Process > Noise > Despeckle, close via Process > Binary > Close, outlier via Process > Noise > Remove Outliers. The resultant image was processed as a binary microglial cell image, with background noise eliminated, and microglial cells were isolated for skeleton analysis. The quantity of microglial cells was quantified by analyzing particles within a 40-infinite area size. ROImanage tool used to amalgamate all microglial cells as “594.” After duplicating the backup image, ROI594 designated and edit/clear outside was used to eliminate the background and preserve microglial cells. Images were saved for examination. Subsequently, binarization and skeletonization were used to generate skeletized images. The Skeleton plugin was used to determine the number of branches and endpoints of microglial cells throughout the BLA region.

### 2.15 Data statistics and graphics methods

We conducted a comprehensive analysis of the collected data using SPSS 29.0, a reliable statistical software, and presented the data visually with GraphPad Prism (version 9.0). Scientific and strict statistical principles were followed in handling quantitative data. Data are presented as the mean ± SEM for precision. Independent-sample t-tests were used for pairwise comparisons, and the LSD test or Bonferroni correction was applied for multiple group comparisons under normal distribution and variance F-test assumptions. Tamhane’s test or the Kruskal-Wallis test was used for multiple group comparisons when the F-test assumptions were not met. Statistical significance was set at *p <* 0.05, for pairwise comparisons, and *p <* 0.01, for multiple group comparisons.

## 3 Results

### 3.1 EA treatment improved social isolation-induced anxiety-like behavior

**Figure 1:**
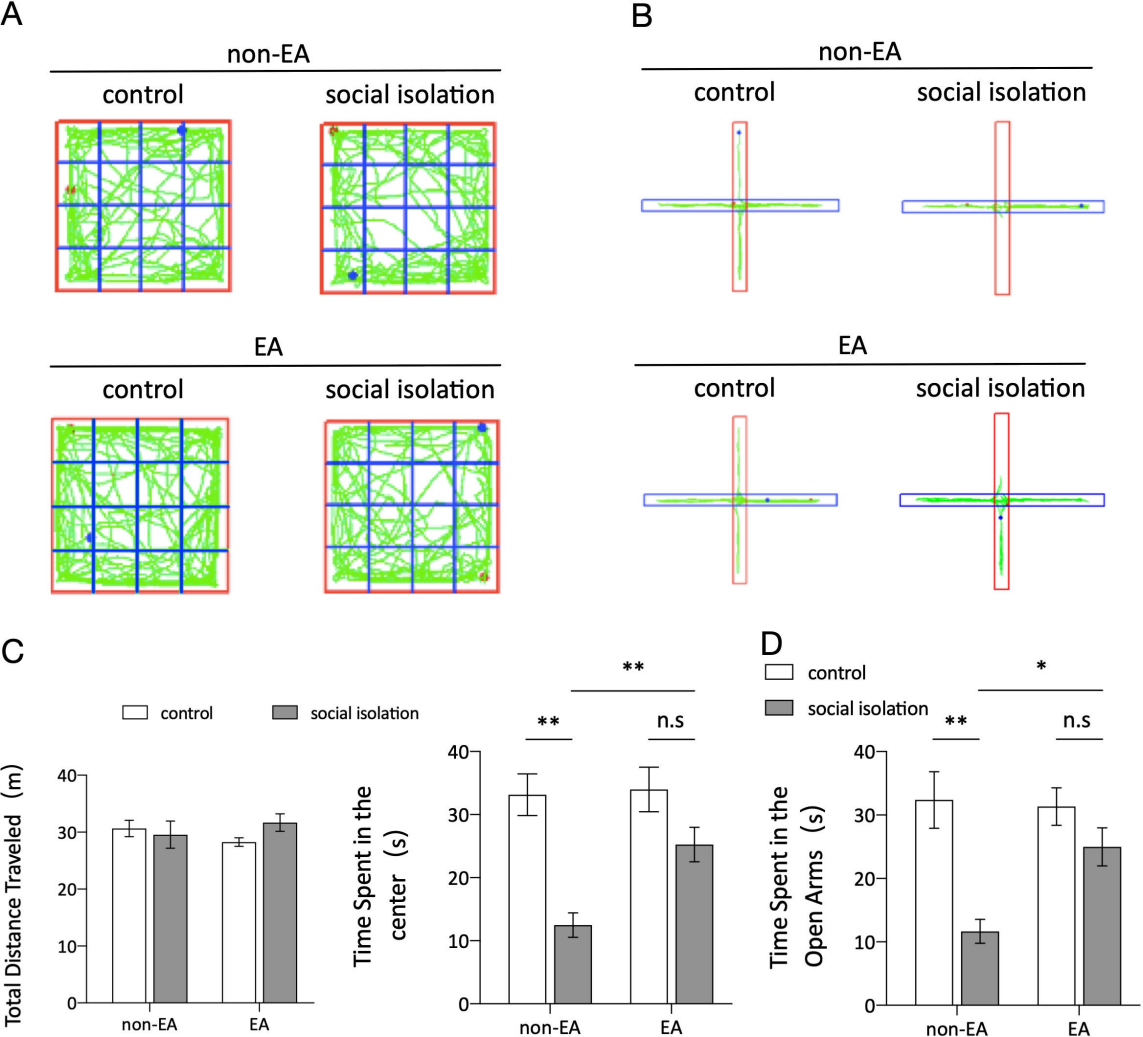
EA treatment improved social isolation-induced anxiety-like behavior. **(A)** Graphical representation of activity traces in each group of mice during the open field test (OFT). **(B)** Tracking of activity patterns in each group of mice in the elevated plus maze (EPM). **(C)** The total distance traveled in the open field test (OFT) and the total time spent by grouped mice in the central area. The results of the two-way ANOVA indicated no significant main effect of EA (F (1, 44) = 0.0074, *p =* 0.9318) or SI (F (1, 44) = 0.5093, *p =* 0.4792) on the total distance traveled in the OFT. Additionally, there was no significant interaction between EA and SI (F (1, 44) = 1.942, *p =* 0.1705). In contrast, the analysis of time spent in the center revealed a significant main effect of EA (F = 24.92, *p <* 0.001) and SI (F = 5.338, *p =* 0.0256) and a significant interaction between the two factors (F = 4.124, *p =* 0.0483) in a sample size of n=12. The results suggest that EA had a significant impact on the time spent in the center, whereas social isolation did not have a significant effect. Total time spent on the EPM open-arm activity. EA×SI two-way ANOVA: main effect of EA, F (1,44) =17.89, p<0.001; main effect of SI, F (1,44)=3.696, p=0.061. Interaction: F (1,44) =5.051, p=0.0297; n=12; ns: not significant. *p < 0.05, **p < 0.01. **(D)** Total time spent on EPM open-arm activity. EA×SI two-way ANOVA: main effect of EA, F (1,44) =17.89, P<0.001; main effect of SI, F(1,44)=3.696, P=0.061. Interaction: F (1,44) =5.051, P=0.0297; n=12; ns, not significant.

During the behavioral testing phase, two widely used methods, the OFT and EPM, were employed to evaluate anxiety-like behaviors. The results of the OFT **Figures 1A, C** indicated no significant variance in the total distance traveled by the mice in the open-field box across the groups, suggesting no discernible impairment in locomotor activity. However, there were notable discrepancies in the time spent in the central area between the control and SI groups, as well as between the SI and SI+EA groups. The results of the OFT and EPM tests demonstrated that SI led to an increase in anxiety-like behavior, whereas EA improved anxiety-like behavior in mice. Specifically, the results **Figures 1B, D** indicated that SI significantly decreased the time spent in the open arms after four weeks, whereas EA treatment led to a significant increase in the number of mice entering the open arm. Collectively, these results suggest that EA intervention may effectively ameliorate anxiety-like behaviors induced by SI.

### 3.2 EA reduced oxidative stress in the amygdala caused by social isolation

**Figure 2:**
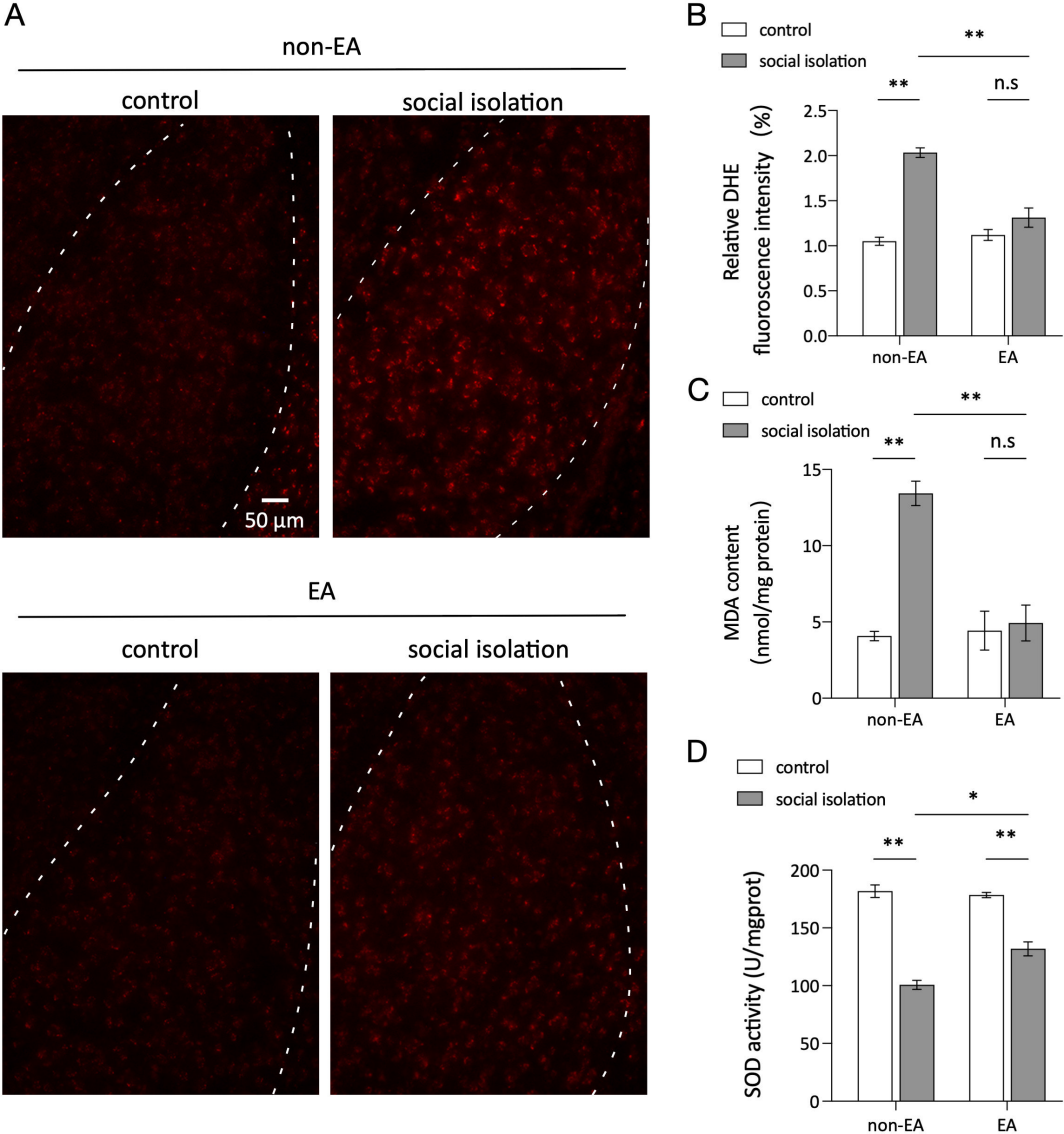
EA reduced oxidative stress caused by social isolation in the amygdala. **(A)** DHE fluorescence intensity in the amygdala region of the brain for each mouse group. **(B)** Mean fluorescence density in the amygdala for each group. Two-way ANOVA results: main effect of EA: F(1,8) =20.93, p=0.0018; main effect of SI: F(1,8) =68.58, *p <*0.0001; interaction effect: F(1,8) =30.91, *p =*0.0005; n=3 ns: not significant. *p < 0.05, **p < 0.01. **(C)** MDA content in the amygdala of each mouse group. Two-way ANOVA results: main effect of EA, F(1.8) =17.79, *p =*0.0029; main effect of SI, F(1,8) =26.09, *p =*0.0029; interaction effect, F(1,8) =21.01, *p =*0.001; n=3; n.s, not significant. **(D)** Statistics of SOD content in the amygdala of each mouse group. Two-way ANOVA: Main effect of EA: F(1,8) =8.825 p =0.0179; Main effect of SI:F(1,8) =186.8 p <0.0001; Interaction effect: F(1.8) =13.58, p =0.0062 n=3; ns, not significant. *p < 0.05, **p < 0.01.

Oxidative stress in the amygdala is intricately linked to anxiety, with alterations in reactive oxygen species (ROS) levels, such as MDA and SOD, playing a significant role in this response (48). The differences and statistical outcomes of DHE staining in the various groups, as depicted in **Figures 2A, B**, illustrate that the level of ROS was notably higher in the SI group than in the control group, and EA intervention effectively mitigated the oxidative stress induced by SI in the amygdala. Additionally, **Figures 2C, D** show the corresponding fluctuations in MDA and SOD levels, which serve as crucial biomarkers of oxidative stress reactions. In the experimental group subjected to social isolation (SI), malondialdehyde (MDA) levels were elevated, and superoxide dismutase (SOD) content was reduced. Conversely, treatment with EA resulted in decreased MDA levels and increased SOD content, aligning with the disparities in fluorescence density observed in DHE staining across the various groups. These congruent findings suggest that SI induces heightened oxidative stress in the amygdala, whereas EA intervention ameliorates the oxidative stress induced by SI.

### 3.3 EA inhibits oxidative stress responses in microglia linked to the basolateral amygdala

**Figure 3:**
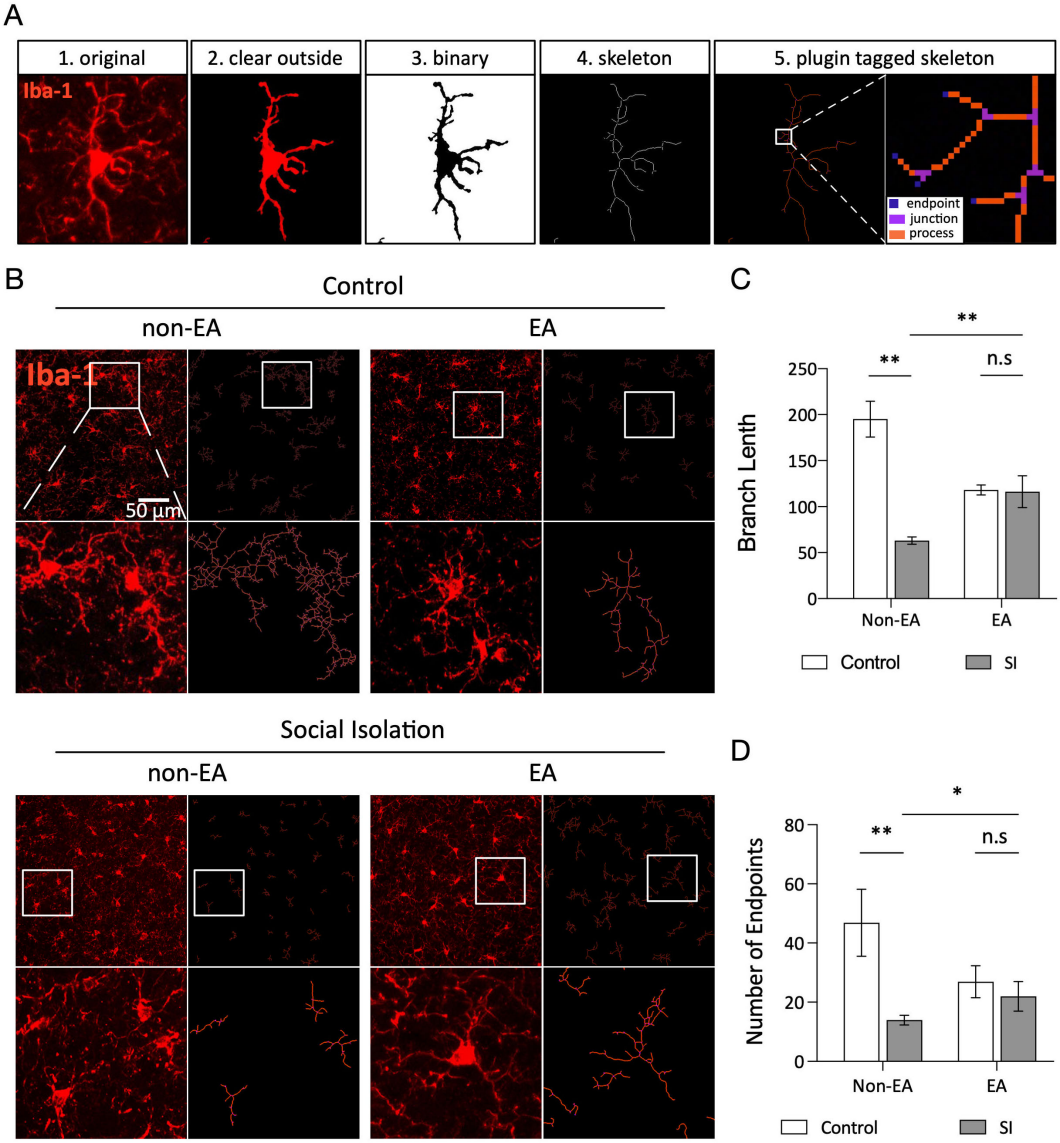
EA ameliorates abnormal microglial morphology in the BLA induced by social isolation. **(A)** Bone analysis of the micrographs. All bone analyses were performed on full-size micrographs (scale =50 µm) using a series of uniform ImageJ plug-in protocols, as detailed in the Methods section. The rules for identification and marking are as follows: the branch lengths are in orange, the endpoints are in blue, and the branch points are in purple. The labeled data were analyzed, summarized, and output. **(B)** Original microglia and skeletonized images of the BLA region in each group of mice. **(C, D)** Statistical analysis of the mean branch lengths and endpoints in the BLA region involved quantification of the number of microglia normalized per skeletal data analysis field. This analysis calculated the branch lengths per cell and endpoints per cell to assess morphological changes in microglia within this region. Statistical analysis also revealed a significant interaction effect of branch length between SI and EA (2way ANOVA: SI: F (1,38) =24.65, *p* <0.0001; EA: F (1,38) =0.7935, p=0.3786; Interaction: F (1,38) =23.33, *p* <0.0001). Interaction of endpoints between SI and EA (2way ANOVA: SI: F (1,38) =9.333, p=0.0041; EA: F (1,38) =0.9299, p=0.3410; Interaction: F (1,38) =5.089, p=0.0299). ns, not significant. *p < 0.05, **p < 0.01.

Overexpression of NOX2 in microglial cells of the amygdala is associated with anxiety (38). Immunohistochemical analysis using antibodies against IBA1 and gp91 confirmed the presence of NOX2 in these cells. The SI group exhibited a notable increase in the number of IBA1-positive and gp91-positive plaques compared to the controls, whereas the EA + SI group demonstrated a decrease in NOX2 expression following EA intervention. These results suggest that social isolation induces elevated NOX2 expression in microglial cells within the amygdala-BLA complex, which can be attenuated by EA. To confirm the heightened NOX2 levels in the amygdala of socially isolated mice due to anxiety, an enzyme-linked immunosorbent assay (ELISA) was employed to quantify NOX2 content (**Figure 3**). Consistent outcomes were observed across the three separate assessments, indicating that EA effectively suppressed NOX2-mediated oxidative stress in the AMY-BLA complex.

### 3.4 EA ameliorates abnormal microglial morphology in the BLA induced by social isolation

**Figure 4:**
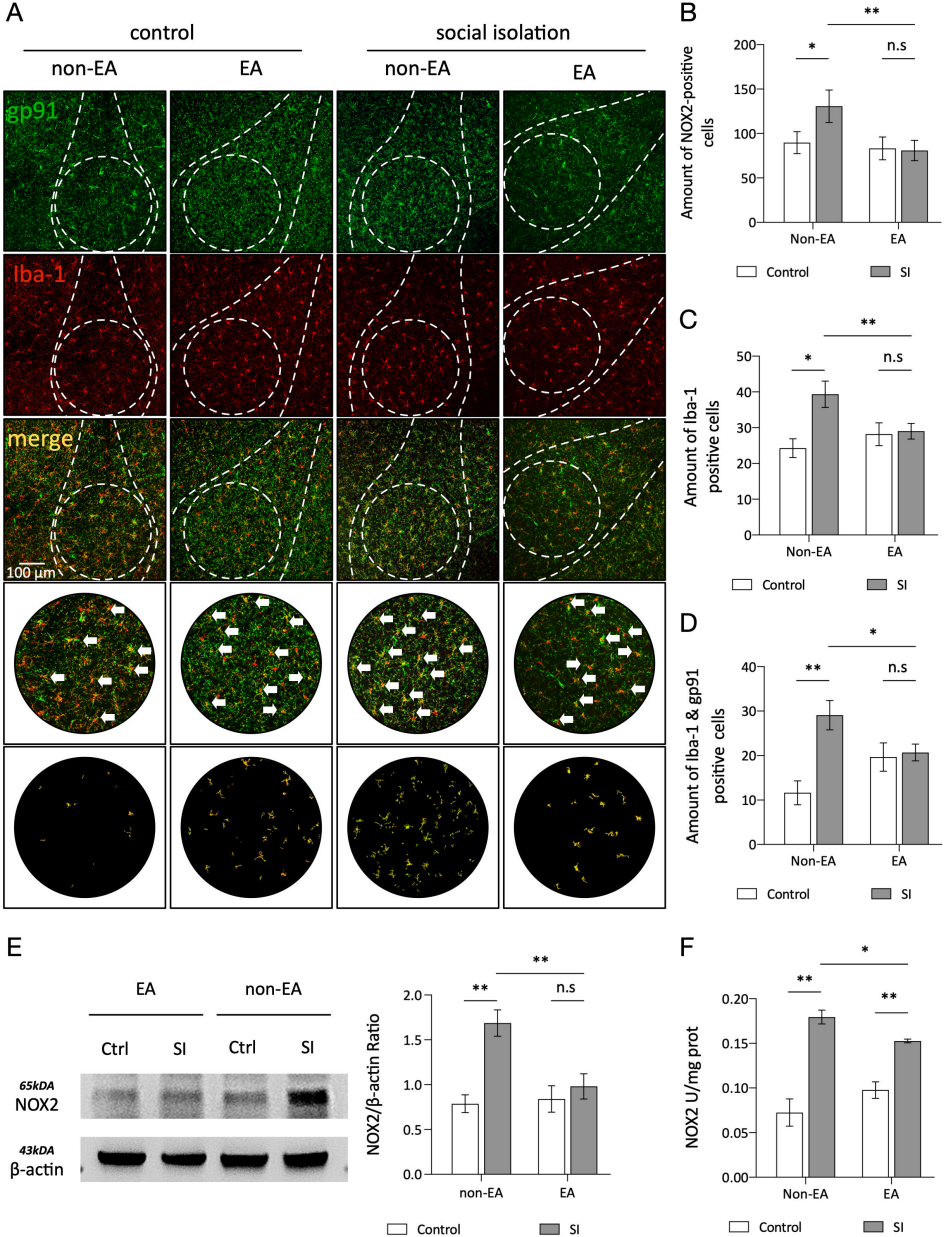
EA inhibits oxidative stress responses in microglia linked to the basolateral amygdala. **(A)** Confocal stack three-dimensional rendering showing NOX2 (green) and IBA1 (red) in the BLA region of the amygdala. White arrows indicate colocalization. Scale bar, 100µm. **(B-D)** Quantitative analysis of IBA1 puncta density (d), NOX2 puncta density, and IBA1-NOX2 colocalization density (n=3 mice per group, one-way ANOVA). Representative protein blots for NOX2 and β-actin are shown. N=1/Group. **(E)** Quantitative analysis of the protein expression ratios for NOX2 and β-actin F. Two-way ANOVA results: Main effect of EA: F(1,8)=5.845 *p* =0.0420 The Main effect of SI: F(1,8)=14.81, *p* =0.0049; Interaction effect: F(1,8)=7.910, *p* =0.0228; n=1 ns: not significant. *p < 0.05, **p < 0.01. **(F)** ELISA measurements of total NOX activity in each group. Two-way ANOVA results: Main effect of EA: F(1,8)=0.0072 *p* =0.9345 The Main effect of SI: F(1,8)=67.86, *p* <0.01; interaction effect: F(1,8)=6.992, *p* <0.01; n=3, ns, not significant. *p < 0.05, **p < 0.01.

Microglia exhibit a highly branched morphology in their resting state, which undergoes adjustments in response to pathological stimuli by shortening processes and expanding the cell body. These dynamic and acute morphological changes correlate with the intensity of stimulation. Microglia play a crucial role in maintaining cellular homeostasis and responding to external stimuli (46, 47). **Figure 4A** illustrates the process of microglial analysis, whereas **Figure 4B** shows the varying morphologies of microglia in the BLA of different groups. Statistical analysis of branch length and endpoints indicated that microglia in the social isolation (SI) group exhibited shorter branch lengths and fewer endpoints than those in the control group (**Figures 4C, D**). Following EA treatment, the data revealed that the EA + SI group displayed significantly longer branch lengths, more endpoints, and broader spatial exploration of the microglial branches. These findings suggest that social isolation-induced stress leads to extensive debranching of microglia in the BLA and that EA intervention can ameliorate this phenomenon.

## 4 Discussion

Anxiety disorders are becoming increasingly prevalent, particularly among Gen Z, due to rising social pressures and mental health challenges (14). Current pharmacological treatments, though effective, often present significant side effects, and their long-term efficacy remains a concern, this has spurred interest in non-drug therapies, such as electroacupuncture (EA), which has demonstrated potential in treating mental health conditions, including anxiety (7) (8). However, the specific neurobiological mechanisms underlying EA’ s therapeutic effects remain underexplored.

Our study focused on the effects of EA in a social isolation-induced anxiety model in mice, providing new insights into the neurobiological pathways influenced by EA. We found that EA significantly reduced anxiety-like behaviors, as evidenced by increased time spent in the open arms of the elevated plus maze and in the center of the open field test. These behavioral changes were accompanied by notable reductions in oxidative stress markers, including NOX2 and malondialdehyde (MDA), and an increase in superoxide dismutase (SOD) activity. These results suggest that EA exerts its anxiolytic effects, at least in part, by modulating oxidative stress pathways in the BLA, a region critically involved in the regulation of emotions.

Neurotransmitters, such as γ-aminobutyric acid (GABA), norepinephrine (NA), acetylcholine (ACh), serotonin (5-HT), and glutamate (Glu), play a crucial role in regulating the functionality of neural circuits (49). Dysregulation of neurotransmitter systems is closely linked to behavioral alterations, including the development of anxiety (49, 50). Chronic stress has been shown to significantly enhance glutamate release within BLA-associated neural circuits (51, 52), while simultaneously impairing GABAergic inhibitory synaptic transmission (51, 53), thereby disrupting the excitatory-inhibitory balance in these circuits and contributing to the neurobiological mechanisms underlying anxiety.

Recent studies have increasingly implicated oxidative stress as a critical factor in the pathophysiology of anxiety disorders (48). Reactive oxygen species (ROS), key byproducts of oxidative stress, disrupt neuronal function and plasticity through mechanisms such as lipid peroxidation and protein oxidation. These processes contribute to synaptic dysfunction and dysregulation of neurotransmitter systems. Notably, glutamate transmission has been frequently highlighted in studies as a key aspect affected by oxidative stress (38, 54).

Our findings showing that EA treatment reduced ROS production and restored oxidative balance, likely contributing to the observed reduction in anxiety-like behaviors. This is consistent with previous studies highlighting the role of antioxidants like SOD in counteracting oxidative damage and ameliorating anxiety symptoms (55).

EA treatment alleviated oxidative stress and anxiety-like behaviors in this social isolation model. Therefore, research on oxidative stress will further clarify the potential mechanism by which EA improves anxiety-like behavior. Although multiple members of the NADPH oxidase family, which are closely associated with oxidative stress, can generate ROS, NOX2 plays a key role in the specific environment of the central nervous system, particularly in scenarios involving microglial activation and immune responses (37). Many pieces of evidence also suggest that the expression of NOX2 plays a key role in the progression of anxiety (38). Microglia are the main immune cells of the central nervous system and play an important role in neurological diseases, particularly oxidative stress and inflammatory processes (37).

Therefore, we performed immunofluorescence colocalization experiments with microglia and NOX2 to assess their association. It was observed that in the social isolation (SI) group, compared with the control group, the colocalization of microglia and NOX2 was significantly increased. After EA treatment, the increase in co-expression was reduced (**Figure 3**). This result supports a strong association between anxiety and NOX2 protein overexpression in microglia. To investigate the specific association between oxidative stress and microglia, we counted microglia in the control and treatment groups. It was found that in the SI group, the number of microglia increased compared to that in the control group. However, after EA treatment, there was a significant difference between the SI + EA and SI groups. Additionally, there was no significant difference between the control +EA and CTRL groups (**Figure 3C**). EA treatment improved anxiety symptoms and decreased NOX2 expression related to microglial activation, but did not inhibit microglial cell proliferation. Numerous studies have indicated that pathogen- and damage-associated molecular patterns (PAMPs and DAMPs, respectively) including chronic stress can accelerate microglial proliferation (39, 56)^]^. Studies have also suggested that EA inhibits microglial cell proliferation by reducing or eliminating microglial cells, leading to improved cognitive decline related to neuroinflammation (57, 58). However, the study mentioned that the function of microglia is closely associated with their activation phenotype, highlighting the necessity of characterizing activation states to fully understand their functional roles (46). Additionally, a study on Alzheimer’s disease found that despite similar total microglial cell counts in both groups, the treatment group exhibited reduced activation of these cells with anti-inflammatory effects (59). In the experiment of knocking out R47H, which is closely related to microglial activation, it was also observed that the effect of the gene mutation on the number of microglial cells was much less significant (60). Another study on sleep and its association with microglial cells indicated that fragmented sleep may affect microglial activation morphology by regulating the transcriptional activity of specific genes rather than simply changing cell numbers (61).

Overall, these studies emphasize the complex relationship between microglial cell number and their function and activation status. Some studies have suggested that inhibition of microglial cell proliferation may improve neuroinflammation, whereas others have indicated that different pathways regulating gene expression or activation states may affect microglial function, not just by changing the number of cells. We considered the differences in microglial cell numbers between different disease models or brain regions, as well as the reactive changes in microglial cells caused by different interventions, all possible factors influencing the degree of microglial cell activation induced by stress-inducing factors.

The number and morphology of microglia are closely linked to various neurological diseases, with microglial morphology being a key indicator of their activation status (46). Therefore, Through colocalization experiments, we observed that microglia were activated in response to chronic stress, and this activation was associated with changes in both the number and morphology of microglia. Based on these observations, we hypothesize that chronic stress leads to an increase in the number of microglia, while EA therapy alleviates anxiety symptoms primarily by inducing adaptive morphological changes in microglia, rather than solely relying on inhibiting their proliferation. To test this hypothesis, we conducted a morphological skeletal analysis of microglia from different experimental groups. Skeleton analysis revealed that CMS significantly reduced both the branch length and the number of endpoints of microglia. However, EA therapy significantly increased these parameters in mice subjected to SI, indicating that EA-induced morphological changes are not merely fluctuations in cell quantity (**Figures 4C, D**). Instead, EA therapy appears to trigger adaptive changes in microglial morphology, suggesting a more refined modulation of microglial function that goes beyond proliferation control (**Figure 4B**). Importantly, these changes do not imply that microglial proliferation is entirely excluded; rather, the primary mechanism of action of EA therapy seems to involve modulating microglial morphology to enhance immune monitoring without excessive activation, as would be seen in pathological conditions. While, and unexpectedly, in mice not exposed to SI, we observed that EA therapy significantly reduced microglial branch length (**Figure 4C**) and the number of endpoints (**Figure 4D**). These changes in healthy mice may reflect a mild immune response induced by acupuncture treatment, but without pathological consequences, as no significant behavioral changes were observed (**Figure 1**). In contrast, for the two groups subjected to social isolation, EA therapy further enhanced the SI-induced microglial changes, including increased branching and reduced cell numbers, and was associated with a significant reduction in anxiety-like behaviors. Consistent with previous literature reports (62), and in contrast to the typically unidirectional regulatory effects of pharmacological agents, EA has occasionally been shown to exert bidirectional influences. Our results suggests that EA therapy may promote neuroimmune homeostasis by enhancing microglial surveillance and local neuroenvironmental regulation. This morphological adjustment improves the immune monitoring capacity, enabling efficient resource allocation and cell regulation. Our results are consistent with several previous studies that demonstrate the neuroprotective role of ramified microglia in safeguarding neuronal function (63).

Overall, the comparison between the control and anxiety groups highlights that EA therapy primarily promotes adaptive morphological changes in microglia, which are associated with improved immune monitoring capacity and reduced anxiety-like behaviors. While changes in microglial number and morphology were observed in both groups, these changes were more pronounced in the anxiety group, where EA therapy likely helped restore balance to the immune system and alleviate anxiety symptoms.

Microglia are activated by social psychological stress, which is associated with inflammation and oxidative stress. However, the mechanism of communication between oxidative stress, inflammation, and neural tissue cells during stressful events has not yet been clearly identified. Oxidative stress can not only directly affect nervous system function but may also exert its effects through other pathways (35, 48).

Oxidative stress can activate inflammatory pathways in the nervous system. Immune cells such as microglia may release inflammatory cytokines under the influence of oxidative stress, affecting the function of the nervous system and leading to the emergence of anxiety-like behavior (35, 36). When oxidative stress occurs due to prolonged or severe stress, such as social isolation, it increases through the activation of the HPA axis stress response system. This produces stress hormones, such as cortisol, leading to an excess of free radicals in the body. These free radicals attack various parts of neurons, causing synaptic dysfunction that affects neurotransmitter balance and neural network operations (34, 35). Therefore, whether oxidative stress acts as a cause or result leads to anxiety-like psychological stress, and chronic stress further exacerbates oxidative stress and inflammation (64). Oxidative stress, inflammation, and anxiety-like states form a vicious cycle that worsens anxiety symptoms and may lead to other health problems (65).

EA shows promise as a beneficial effect for anxiety disorders, especially for individuals who do not respond well to pharmacological treatments, and it also has fewer side effects. Our findings provide a theoretical basis for the use of EA in treating anxiety disorders at the animal level. However, this study cannot fully represent the pathophysiological conditions in humans and did not observe the effects of oxidative stress in the amygdala on anxiety-related neuronal function at the electrophysiological level. Additionally, we did not assess neuronal or synaptic activity at specific sites, nor did we evaluate changes in neurotransmitter levels. Future studies will focus on elucidating the effects of EA and oxidative stress on anxiety-related neural mechanisms, with particular attention to synaptic function and neurotransmitter dynamics. Furthermore, we will investigate the long-term impact of EA on anxiety and other stress-related disorders to better understand its therapeutic potential.

## 5 Conclusion

This study provides novel insights into the role of microglial cells in anxiety, demonstrating that EA modulates both the morphology and function of microglia in the BLA. Our findings show that EA reduces oxidative stress and alleviates anxiety symptoms by improving microglial antioxidant status and reducing ROS levels. This study is one of the first to analyze microglial morphological changes in the context of anxiety, highlighting the critical role these immune cells play in regulating anxiety-related behaviors under chronic stress conditions.

**Figure 5:**
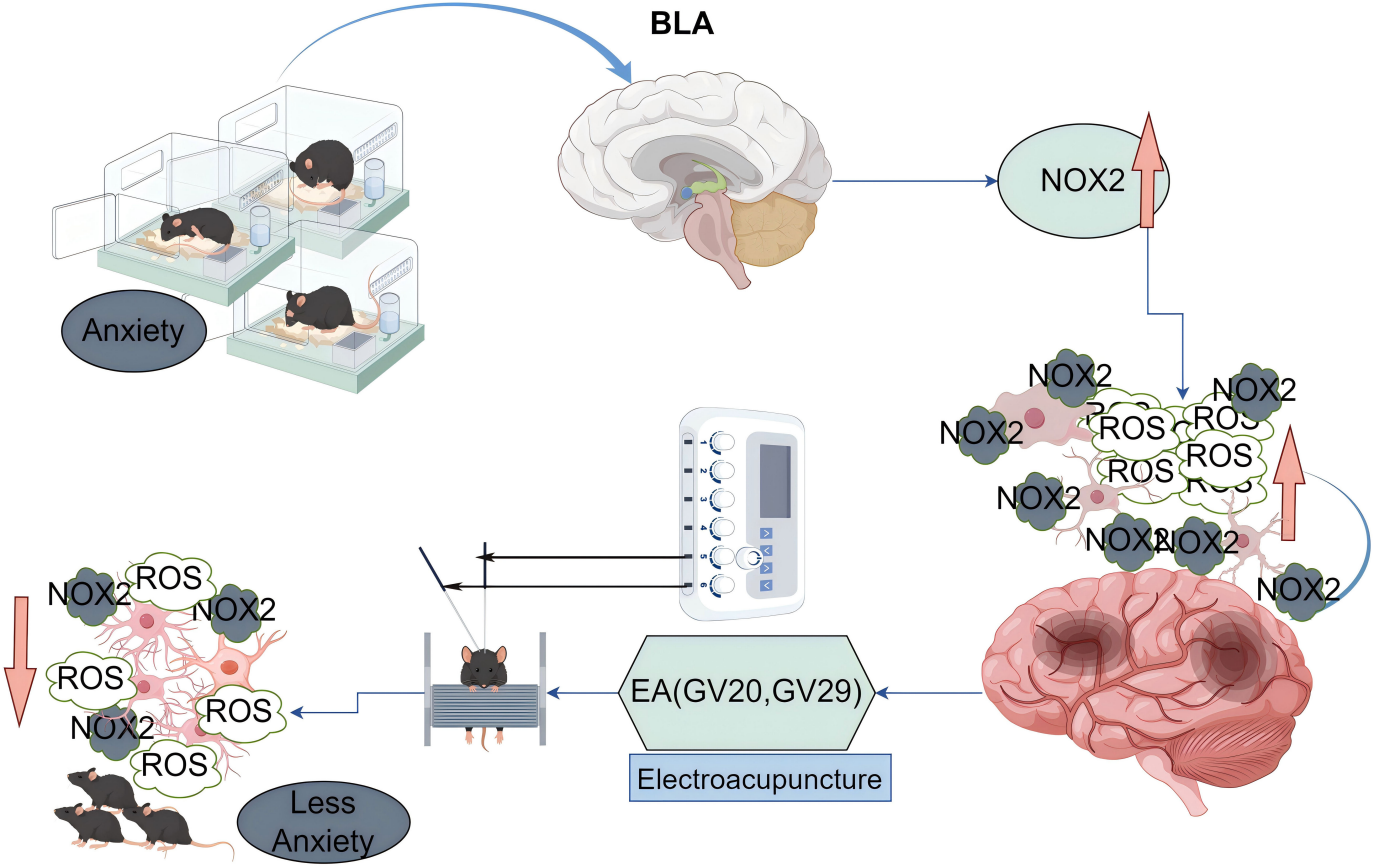
Proposed mechanism of electroacupuncture in modulating oxidative stress and microglial activity induced by social isolation.

The proposed mechanism, as depicted in **Figure 5**, illustrates how EA may exert its anxiolytic effects through the modulation of microglial activity and oxidative stress. The figure shows that social isolation leads to an increase in ROS production, which in turn activates microglia, causing them to adopt an amoeboid morphology associated with a pro-inflammatory phenotype. This activation is linked to the exacerbation of anxiety symptoms. Conversely, EA application is shown to reduce ROS levels, leading to a shift in microglial morphology towards a more ramified state, indicative of a neuroprotective phenotype. This change in microglial status is associated with a reduction in anxiety behaviors, suggesting that EA’s effects on microglia are a key mediator of its anxiolytic action.

Further research is needed to elucidate the specific molecular pathways through which EA modulates microglial activity and to explore the therapeutic potential of targeting microglia in anxiety disorders. Despite the limitations of animal models, these findings offer a promising foundation for developing new treatment strategies for oxidative stress-related neuropsychiatric conditions, particularly those involving microglial dysfunction.

## Data Availability

The original contributions presented in the study are included in the article/**Supplementary Material**. Further inquiries can be directed to the corresponding authors.
