# Supplementary figures and images for: Confronting the anxiety of Generation Z: electroacupuncture therapy regulates oxidative stress and microglia activity in amygdala-basolateral of socially isolated mice
Source: Front Psychiatry. 2025 Feb 6;15:1496201. doi: 10.3389/fpsyt.2024.1496201 (PMC11839672; doi:10.3389/fpsyt.2024.1496201)

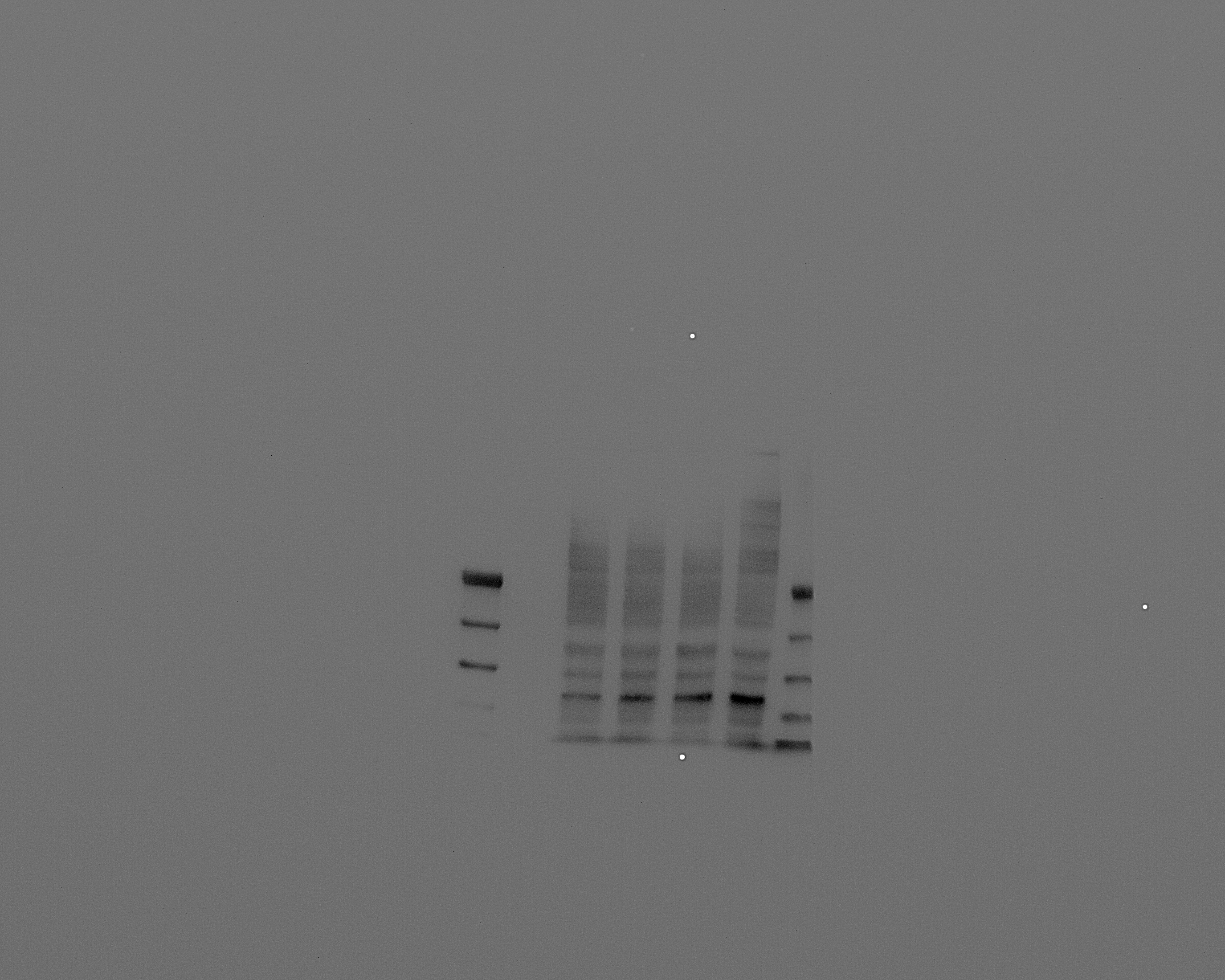

Supplement: Supplementary file 1 [file DataSheet1.zip › ╘¡═╝/230722NOX2╘¡═╝╡┌4╒┼.tif]

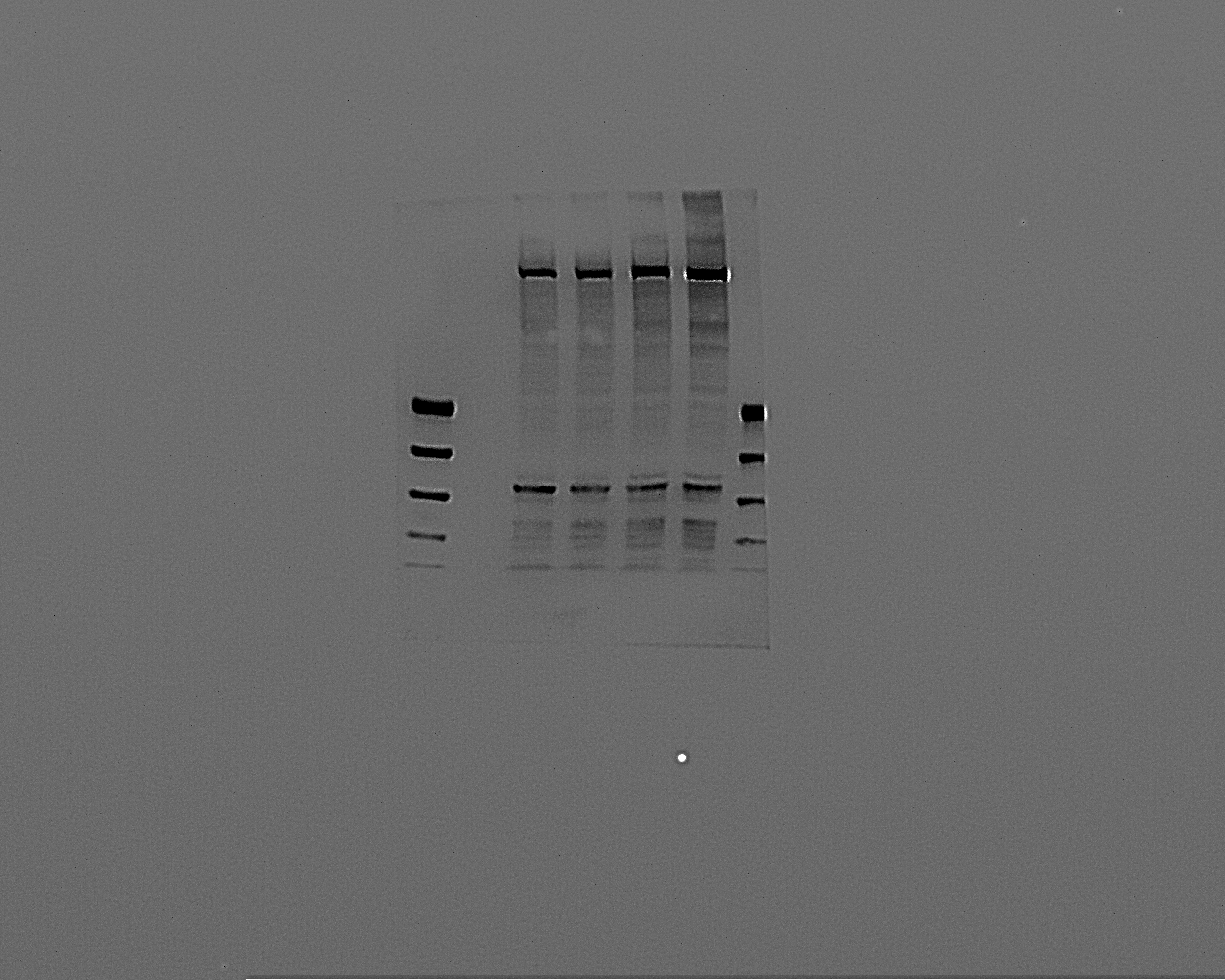

Supplement: Supplementary file 1 [file DataSheet1.zip › ╘¡═╝/230722─┌▓╬╘¡═╝╡┌4╒┼.tif]

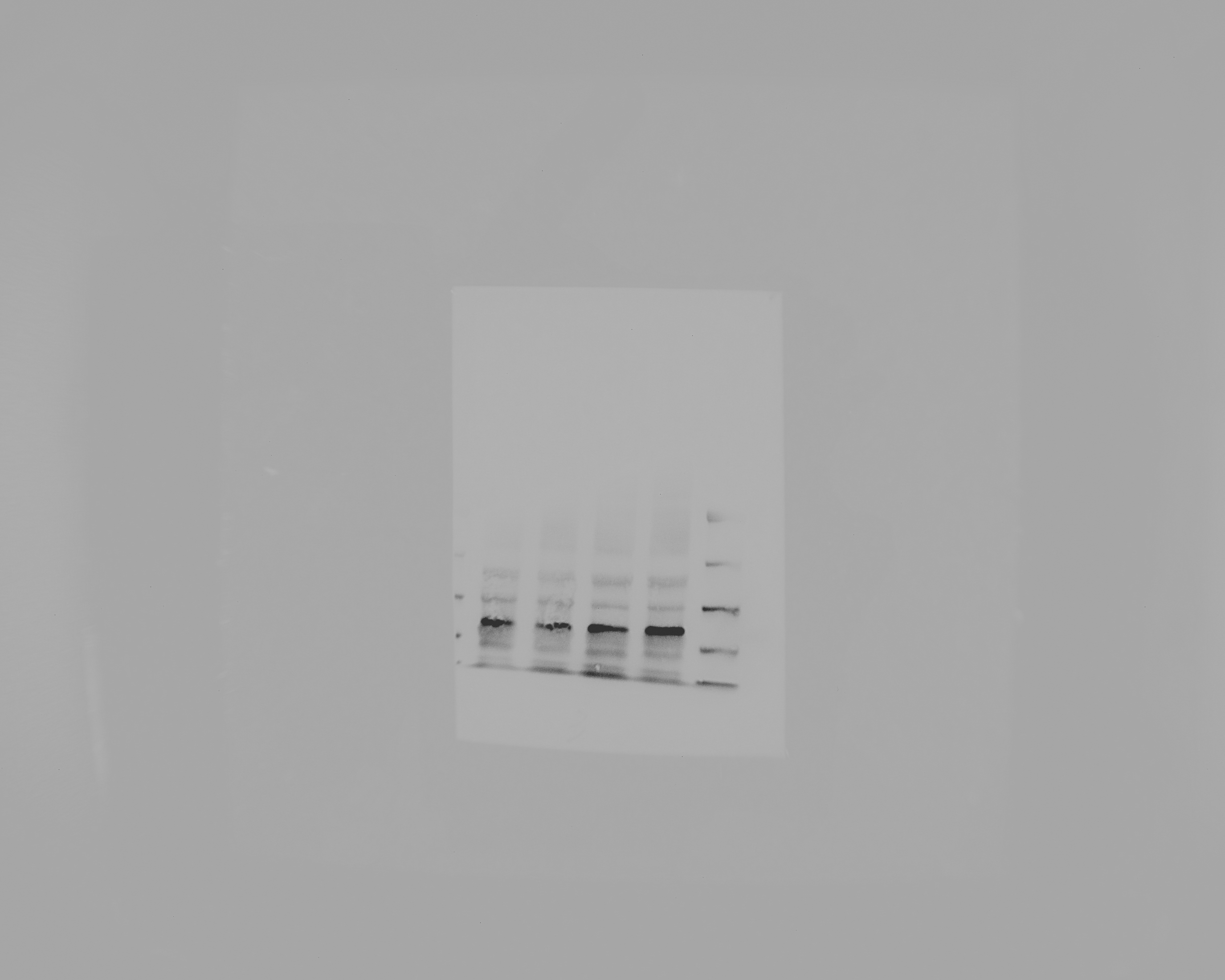

Supplement: Supplementary file 1 [file DataSheet1.zip › ╘¡═╝/230724NOX2╡┌3╒┼.tif]

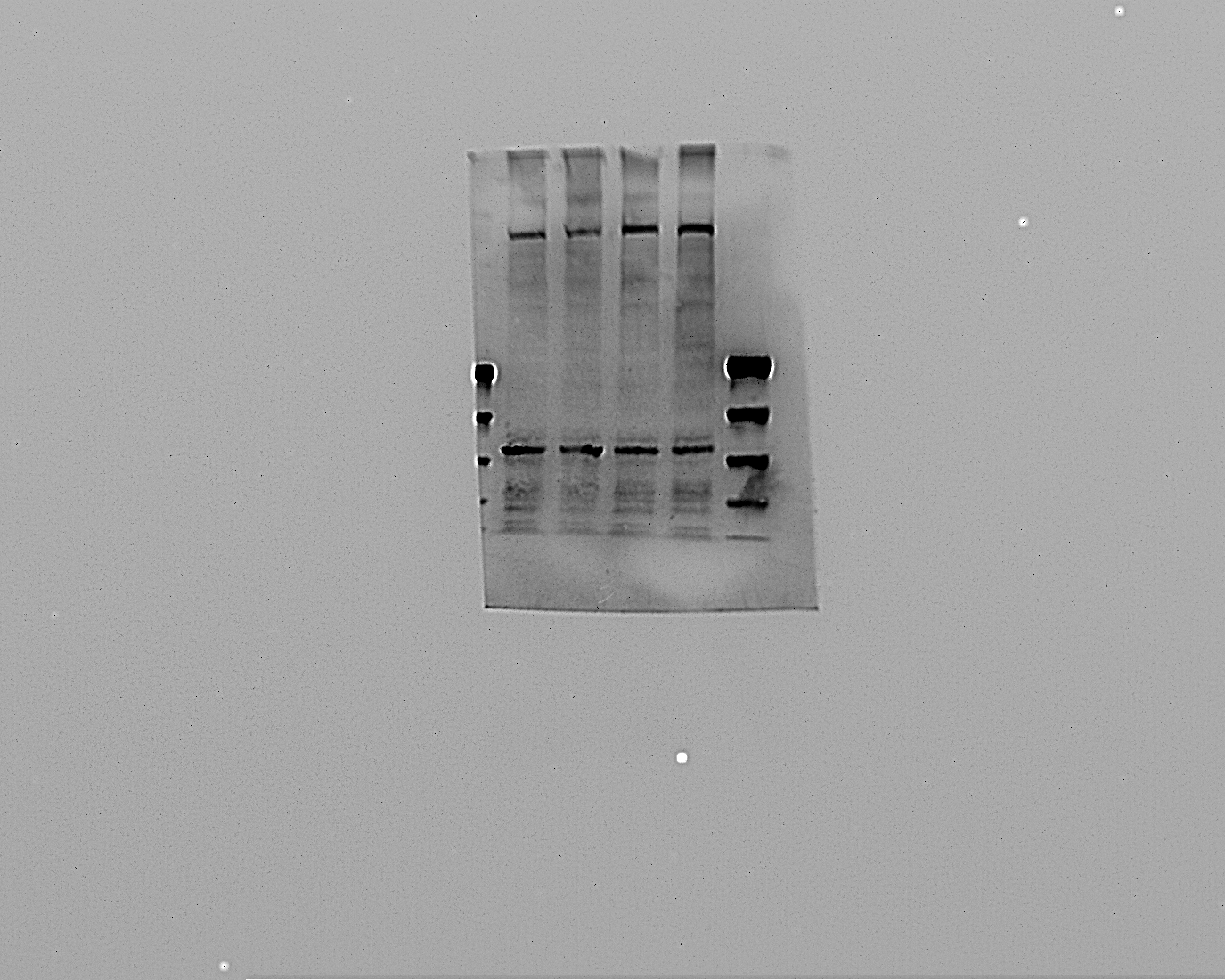

Supplement: Supplementary file 1 [file DataSheet1.zip › ╘¡═╝/230724─┌▓╬-╡┌3╒┼.tif]

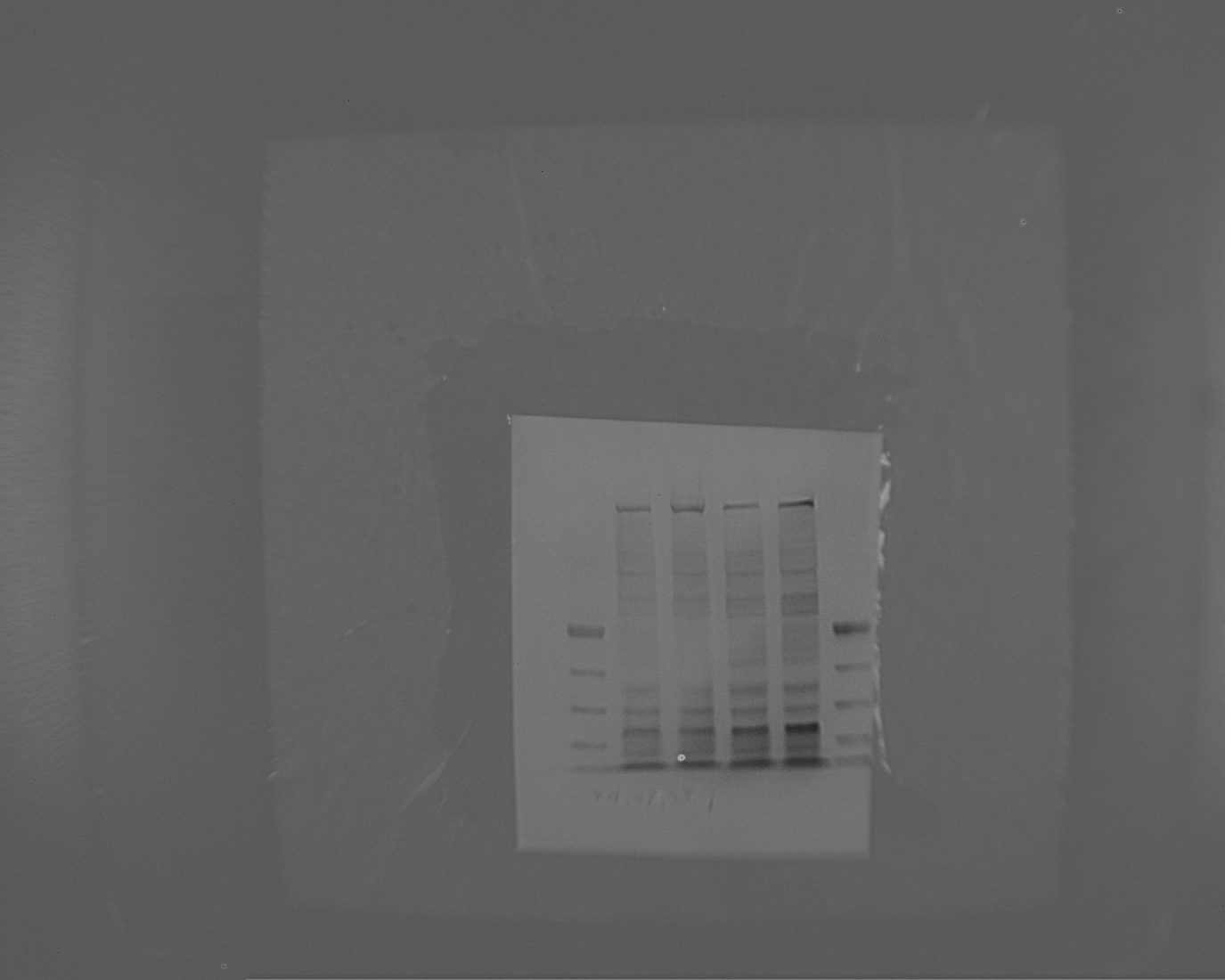

Supplement: Supplementary file 1 [file DataSheet1.zip › ╘¡═╝/230726NOX2╡┌1╒┼.tif]

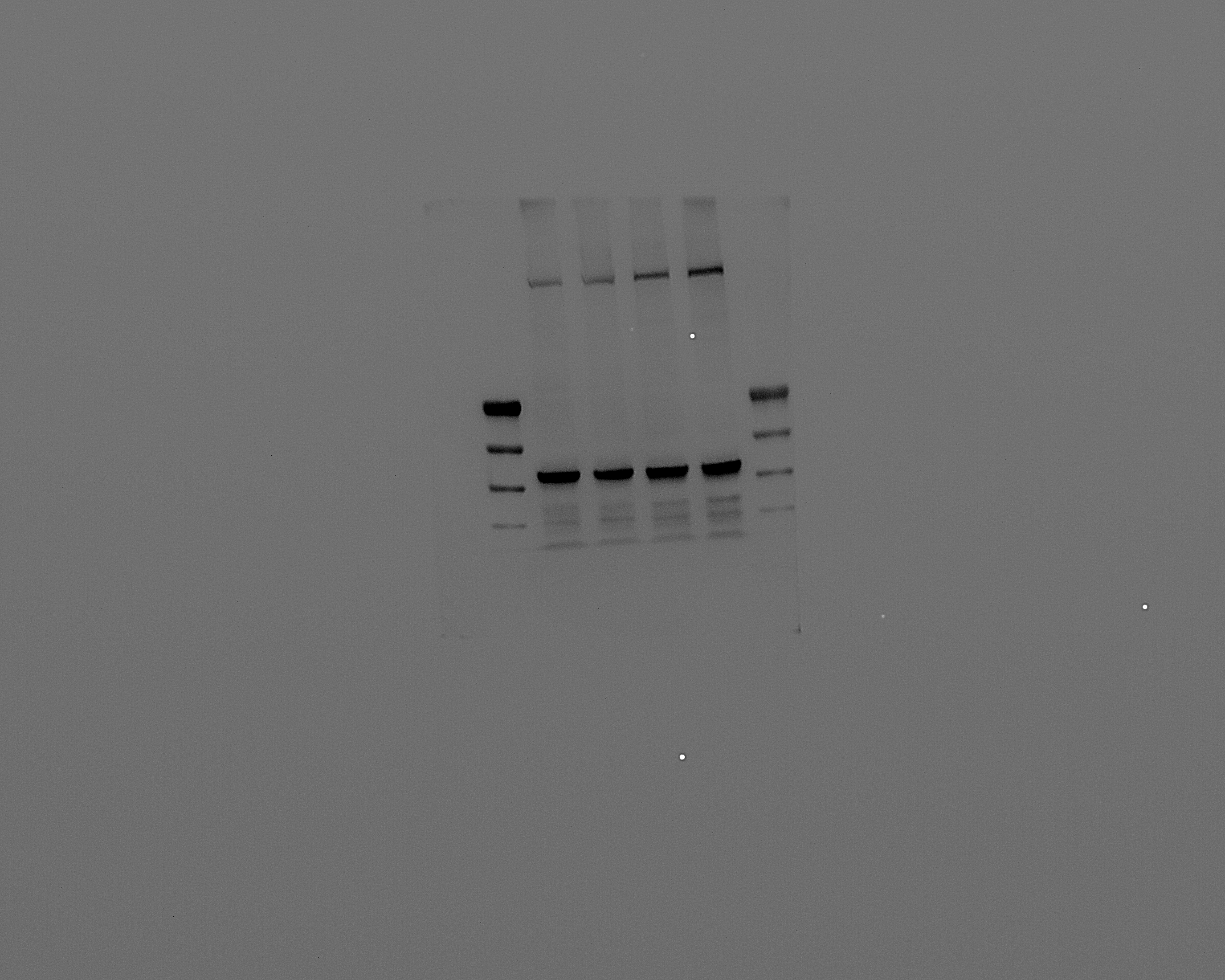

Supplement: Supplementary file 1 [file DataSheet1.zip › ╘¡═╝/230726─┌▓╬╘¡═╝╡┌1╒┼1x1.tif]

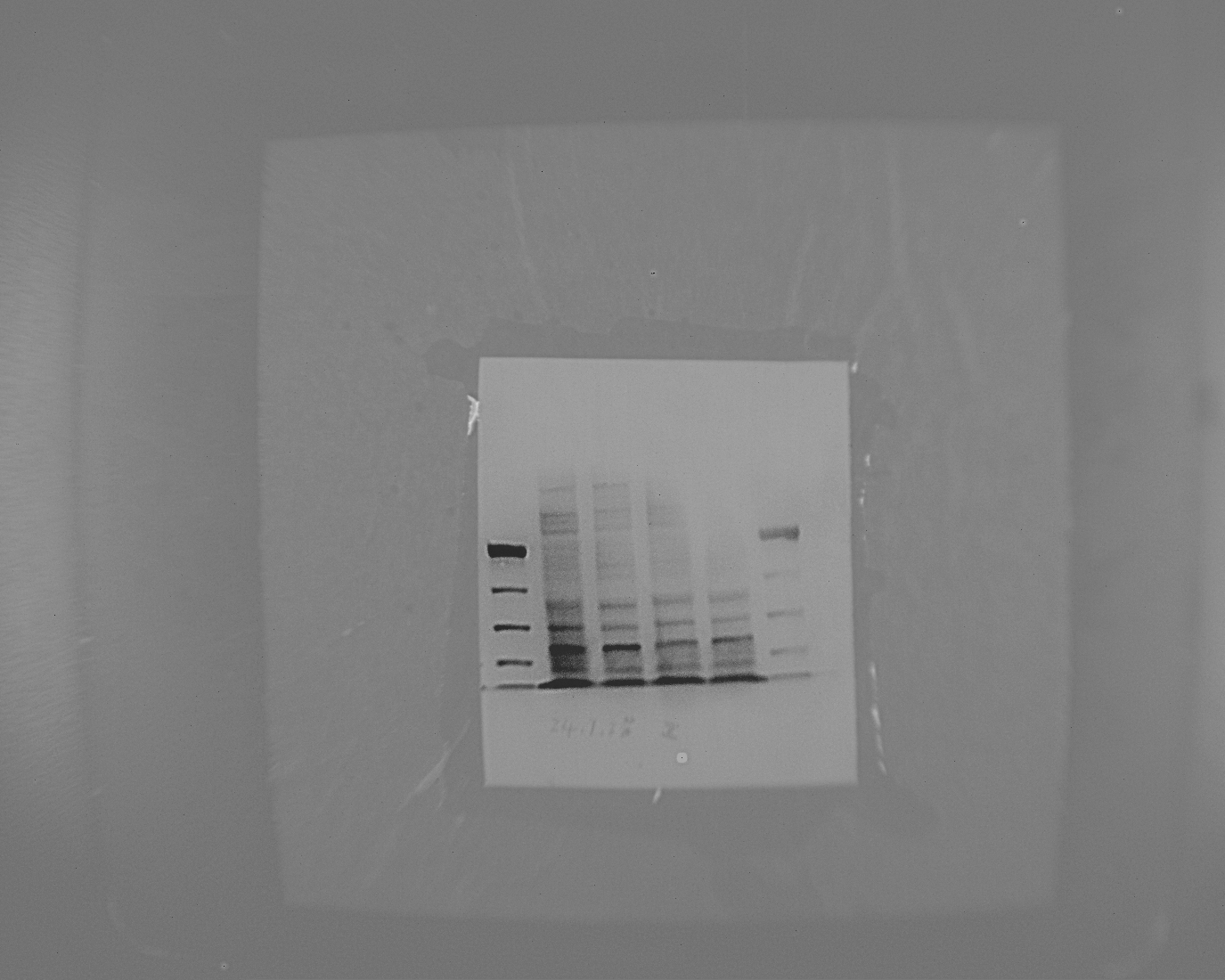

Supplement: Supplementary file 1 [file DataSheet1.zip › ╘¡═╝/230727-NOX2╡┌2╒┼.tif]

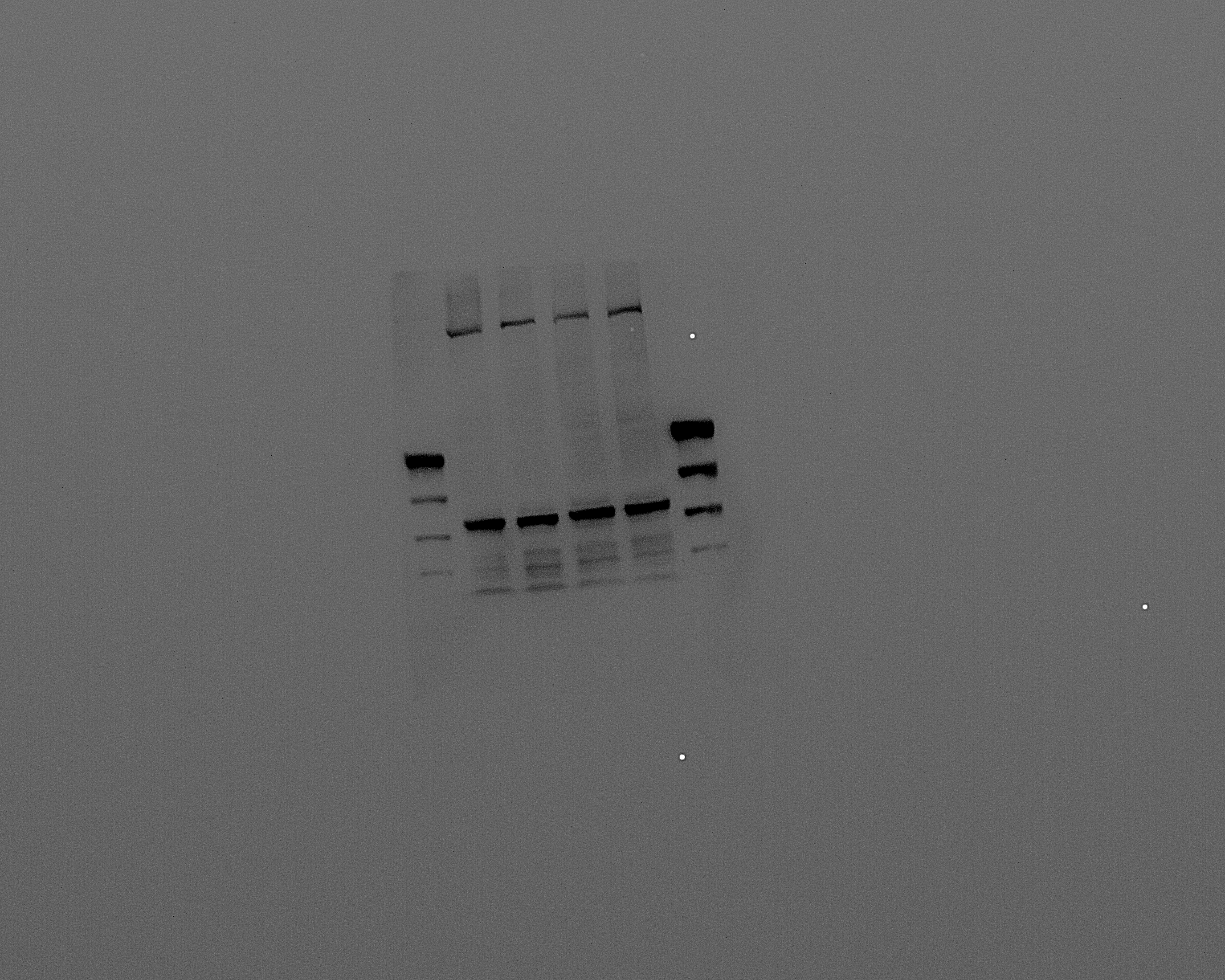

Supplement: Supplementary file 1 [file DataSheet1.zip › ╘¡═╝/230727-─┌▓╬╡┌2╒┼1x1.tif]
